# Commentary: COVID-19 and mental health equity in the United States

**DOI:** 10.3389/fsoc.2020.584390

**Published:** 2020-11-19

**Authors:** Eileen M. Condon, Amanda M. Dettmer, Dylan G. Gee, Cheyanne Hagan, Ka Shu Lee, Linda C. Mayes, Carla S. Stover, Wan-Ling Tseng

**Affiliations:** ^1^Yale School of Nursing, New Haven, CT, United States; ^2^Yale Child Study Center, Yale School of Medicine, New Haven, CT, United States; ^3^Department of Psychology, Yale University, New Haven, CT, United States; ^4^Division of Psychology and Language Sciences, Faculty of Brain Sciences, University College London, London, United Kingdom; ^5^Anna Freud National Centre for Children and Families, London, United Kingdom

**Keywords:** COVID-19, early stress, adversity, developmental psychopathology, long-term strategies

## Introduction

A recent commentary in *Social Psychiatry and Psychiatric Epidemiology* emphasized the disproportionately adverse mental health impacts of the COVID-19 pandemic (Purtle, 2020). While this perspective focused specifically on disparities in financial insecurity and grief stemming from disparities in COVID-19 mortality in adults, we expand this view to incorporate a developmental perspective of the pre-existing inequities experienced by children and families that have been magnified by COVID-19 in the United States.

**Figure 1:**
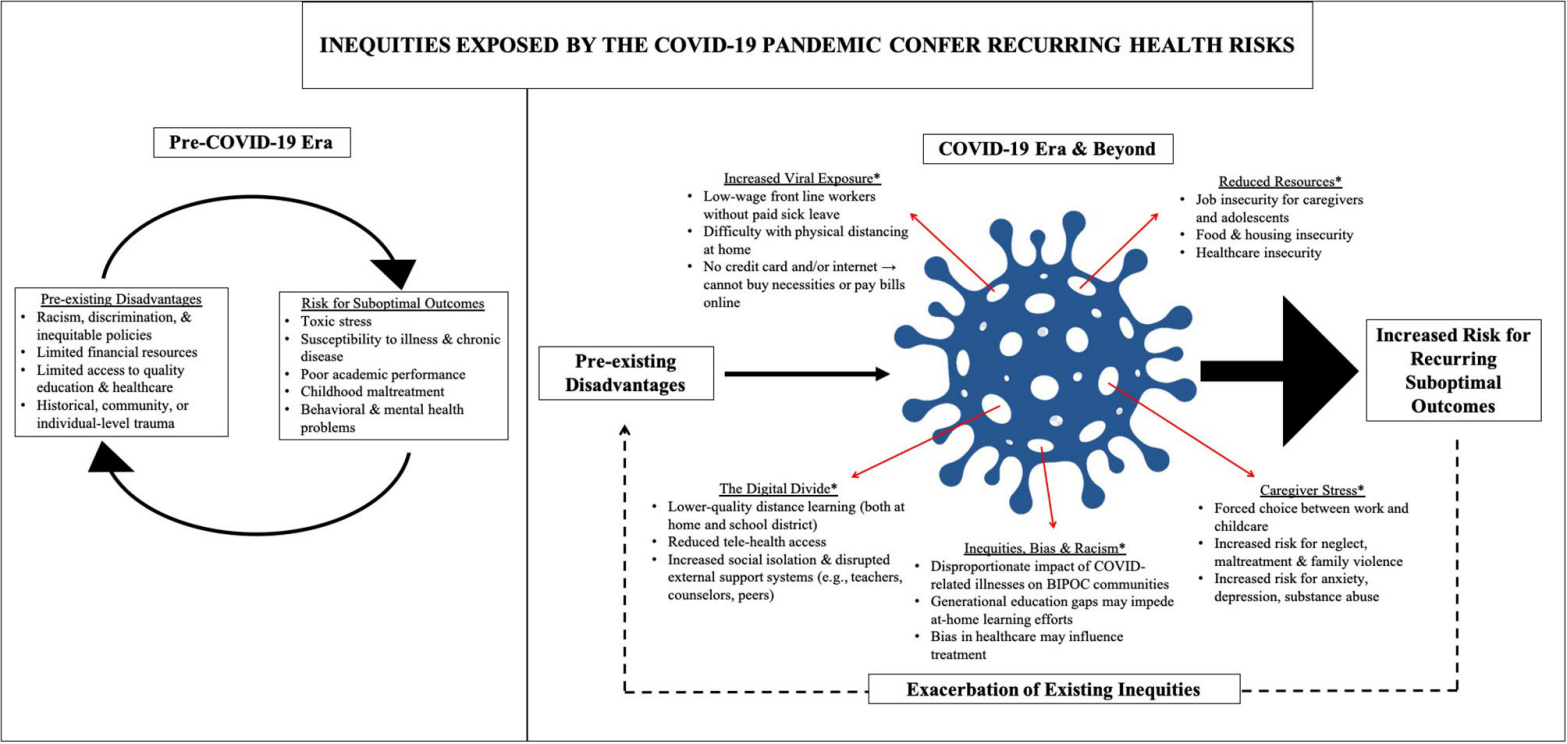
The COVID-19 pandemic magnifies and exacerbates existing inequities (left) for disadvantaged children and families in the United States (indicated by thicker arrows in right), thereby placing them at greater risk for recurring suboptimal health outcomes and exacerbation of existing inequities. The asterisks (*) denote increased risk factors for disadvantaged children and families during COVID-19, which are not mutually exclusive. These are also potential points for intervention.

The COVID-19 pandemic is an abrupt and chronic stressor that puts many children and adolescents at risk for developing mental and behavioral health disorders—particularly those from disadvantaged backgrounds and marginalized communities, as the pandemic has illustrated in the U.S. Before the COVID-19 pandemic, these children were already more likely to experience severe economic hardship, lack access to quality education and other resources critical for coping with adversities, and be at increased risk for maltreatment and exposure to home or community violence, all of which can result in increased risk for adverse health outcomes (Nurius et al., 2015; Jones et al., 2018). Moreover, BIPOC (Black, Indigenous, People of Color) families in the U.S. face systemic racism and discrimination. These inequities are only magnified and perpetuated by the COVID-19 pandemic (Figure 1) (Beaunoyer et al., 2020; Hooper et al., 2020; Turner Lee, 2020a; Van Dorn et al., 2020).

## Toxic Stress—Magnified

A “toxic stress” response occurs when the resources that support children during adversities become taxed or depleted. Caregivers play a central role in buffering stress early in life (Gunnar and Donzella, 2002; Gee et al., 2014), and how caregivers respond to children's stress and convey information about traumatic events has critical effects on children's mental health following trauma (Carpenter et al., 2017). Sudden increases in stress due to the challenges of the COVID-19 pandemic, such as increased viral exposure, even more restricted resources due to increased job, food, housing, and healthcare insecurity, and forced choices between work and childcare, may impair caregivers' ability to provide nurturing, sensitive parenting to help children cope with abrupt lifestyle changes (Cluver et al., 2020; Humphreys et al., 2020; Jain, 2020; Oppel et al., 2020; Power et al., 2020; Van Lancker and Parolin, 2020; Visual Capitalist, 2020).

With long-term major upheavals in daily life and widespread school closures in the U.S., the COVID-19 pandemic is likely to be particularly stressful and compromise the buffering effects of caregiving for families in marginalized or disadvantaged communities. School closures meant that children's support systems outside of the home were disrupted, with less support from teachers and peers, along with disruptions in mental health services provided by schools. For the 22 million children who rely on schools for meals, food insecurity has increased (Bauer, 2020), in part because there was no federal mandate that schools continue to offer food services during closures (Dunn et al., 2020; Van Lancker and Parolin, 2020). Job and housing insecurity have further impacted disadvantaged families (Grinstein-Weiss et al., 2020), and stress may be particularly acute for adolescents who work to support their families. Many caregivers from lower-income families are essential workers (e.g., grocery clerks, sanitation workers, public transportation drivers) whose jobs cannot be done remotely and/or do not offer paid sick leave. Such essential workers thus risk being infected at work or bringing the infection home (Visual Capitalist, 2020). School and daycare closures meant that many caregivers without the means and support for childcare were forced to prioritize job security over health. Existing educational disparities have been and will continue to be amplified for disadvantaged children, who are more likely to lack resources essential to distance learning and have fewer familial resources to support learning at home (Connecticut State Department of Education, 2020; Turner Lee, 2020a,b). Further, material hardship and deprivation may hinder brain development that supports functions such as literacy, problem-solving, decision-making, and emotion regulation, which are important for developing resilience and coping with future stressors (Johnson et al., 2016).

## Discussion

In these ways, the COVID-19 pandemic will have disproportionately long-lasting negative effects on the most vulnerable children and families in the U.S., including those living in poverty or with prior trauma histories, which are strong predictors of risk for mental health disorders (McLaughlin et al., 2012). Of particular concern is increased risk for maltreatment and exposure to family violence (Humphreys et al., 2020). Parenting stress, economic instability, and substance abuse are major risk factors for abuse and neglect (Stith et al., 2009) that are likely to be heightened during the pandemic. Unfortunately, detection of abuse and thus the potential to prevent it or intervene may be substantially impeded due to reduced contact with educators and healthcare providers, two primary sources of referrals to children's services. Though estimates of maltreatment exposure and referrals are not yet available, media and agency reports in the country have noted marked declines in referrals to child protection but increases in calls by minors to domestic violence and abuse hotlines.

Given the vast existing social and economic inequities across societies, it should come as no surprise that an abrupt stressor like COVID-19 has a disproportionate effect on disadvantaged and marginalized children. Pre-existing disadvantages that lead to increased viral exposure, including lack of quality health care, childcare, and paid sick leave, also have downstream consequences that put all individuals at risk (Woolf, 2017). This public health crisis should be taken as a call to action not only to meet the immediate needs of disadvantaged children, but also to support long-term strategies to dismantle the policies and institutional structures that systematically oppress low-income and BIPOC families across the country. Investment in interventions and adoption of policies that build on family strengths, including but not limited to early home visiting, school-based health centers, a living wage, paid family leave, and universal healthcare and childcare, are critical.

The health of a society depends on how well it invests in and cares for its most vulnerable. The COVID-19 pandemic highlights what we already know: we need to do better.

## Author Contributions

All authors contributed equally in writing the manuscript. EC and AD designed the figure.

## Conflict of Interest

The authors declare that the research was conducted in the absence of any commercial or financial relationships that could be construed as a potential conflict of interest.
